# Uptake of services for prevention of mother-to-child transmission of HIV in a community cohort in rural Tanzania from 2005 to 2012

**DOI:** 10.1186/s12913-015-1249-6

**Published:** 2016-01-06

**Authors:** Annabelle Gourlay, Alison Wringe, Jim Todd, Caoimhe Cawley, Denna Michael, Richard Machemba, Benjamin Clark, Clemens Masesa, Milly Marston, Mark Urassa, Basia Zaba

**Affiliations:** 1 Faculty of Epidemiology and Population Health, London School of Hygiene & Tropical Medicine, Keppel Street, London, WC1E 7HT UK; 2 National Institute for Medical Research, Mwanza, Tanzania

**Keywords:** HIV, Prevention of mother-to-child transmission (PMTCT), Pregnancy, Cohort, Antiretroviral drugs, Coverage

## Abstract

**Background:**

Estimates of population-level coverage with prevention of mother-to-child transmission (PMTCT) services are vital for monitoring programmes but are rarely undertaken. This study describes uptake of PMTCT services among HIV-positive pregnant women in a community cohort in rural Tanzania.

**Methods:**

Kisesa cohort incorporates demographic and HIV sero-surveillance rounds since 1994. Cohort data were linked retrospectively to records from four Kisesa clinics with PMTCT services from 2009 (HIV care and treatment clinic (CTC) available in one facility from 2008; referrals to city hospitals for PMTCT and antiretroviral treatment (ART) from 2005). The proportion of HIV-positive pregnant women residing in Kisesa in 2005–2012 who accessed PMTCT service components (based on linkage to facility records) was calculated per HIV-positive pregnancy and by year, with adjustments made to account for the sensitivity of the linkage algorithm.

**Results:**

Out of 1497 HIV-positive pregnancies overall (to 849 women), 26 % (*n* = 387/1497) were not linked to any facility records, 35 % (*n* = 519/1497) registered for ANC but not HIV services (29 % (*n* = 434/1497) were not tested at ANC or diagnosed previously), 8 % (*n* = 119/1497) enrolled in PMTCT but not CTC services (6 % (*n* = 95/1497) received antiretroviral prophylaxis), and 32 % (*n* = 472/1497) registered for CTC (14 % (*n* = 204/1497) received ART or prophylaxis) (raw estimates). Adjusted estimates for coverage with ANC were 92 %, 57 % with HIV care, and 29 % with antiretroviral drugs in 2005–2012, trending upwards over time.

**Conclusions:**

Population-level coverage with PMTCT services was low overall, with weaknesses throughout the service continuum, but increased over time. Option B+ should improve coverage with antiretrovirals for PMTCT through simplified decisions for initiating ART, but will rely on strengthening access to CTC services.

**Electronic supplementary material:**

The online version of this article (doi:10.1186/s12913-015-1249-6) contains supplementary material, which is available to authorized users.

## Background

In 2011, the United Nations Global Plan set out ambitious targets to eliminate mother-to-child transmission of HIV by 2015 [1]. Scale-up of prevention of mother-to-child transmission (PMTCT) programmes has contributed to an estimated reduction of 60 % in new paediatric HIV infections since 2001, although 240,000 infections still occurred globally in 2013 [2]. Most infections via mother-to-child transmission occur in sub-Saharan Africa [2], with 21 of the 22 Global Plan priority countries, including Tanzania, located in the region [1]. Estimates suggest 65 % of HIV-infected pregnant women living in these countries received antiretroviral (ARV) drugs for PMTCT in 2012, falling short of targets for universal coverage [3].

**Fig. 1:**
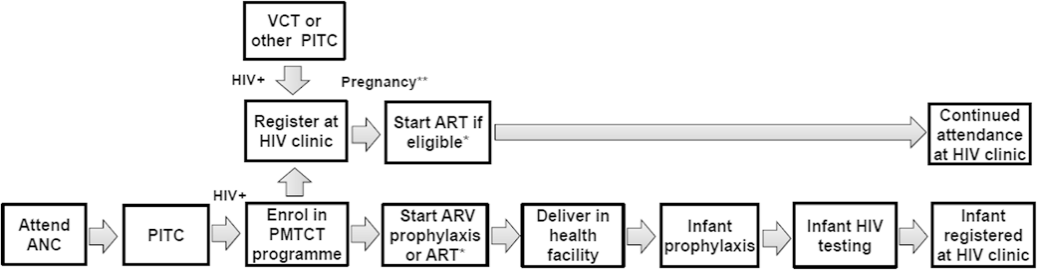
PMTCT cascade of services for mothers and infants. *ANC* antenatal clinic, *ARV* antiretroviral, *ART* antiretroviral therapy, *PITC* provider initiated testing and counselling, *VCT* voluntary counselling and testing. *ARV prophylaxis for PMTCT, or ART for the woman’s own health if she meets eligibility criteria. **Woman was pregnant at the time of VCT or other PITC, or became pregnant after registration at the HIV clinic, before or after starting ART

PMTCT programmes include a ‘cascade’ of services beginning with antenatal clinic (ANC) attendance and provider-initiated HIV testing and counselling (PITC) (Fig. 1). Pregnant women diagnosed with HIV are referred to HIV clinics for long-term care and treatment, provided antiretroviral drugs, advised to deliver in a health centre, and counselled about infant feeding. HIV-exposed infants receive ARV prophylaxis, are tested for HIV, and if diagnosed HIV-positive are enrolled in HIV programmes. Global PMTCT guidelines have evolved from short-course ARV prophylaxis for mothers and infants to prevent HIV transmission, towards longer and more potent ARV regimens with the potential to improve maternal health [4, 5]. The latest guidelines (‘Option B+’) recommend all HIV-positive pregnant women initiate ART for life [4]. Several African countries have adopted this approach, including Tanzania which began implementing Option B+ in 2013 [6].

Estimates of coverage with PMTCT services among all HIV-infected pregnant women are vital to monitor progress relative to targets, and to secure donor funding for PMTCT programmes [7]. Measuring coverage for each component of the cascade can highlight programme weaknesses where a disproportionately high number of women fail to receive PMTCT services. However, national-level PMTCT coverage may be over-estimated due to problems with the quality of aggregated programme data (numerator) and estimating or modelling the number of pregnant HIV-positive women (denominator) [7]. Furthermore, national-level estimates may mask substantial regional differences in coverage, but regional-level estimates are rarely available. Most research investigating uptake of PMTCT components starts from the point of programme entry, missing HIV-infected women who are undiagnosed, or diagnosed but do not seek services [8]. Innovative approaches have been attempted by researchers in West and Southern Africa to measure coverage with PMTCT prophylaxis (nevirapine) using umbilical cord blood samples, but these cross-sectional surveillance studies were also limited to women who attended health services for delivery [9].

Linkage of community research data to routinely collected clinic data provides an important opportunity to calculate direct estimates of coverage with PMTCT services, yet very few studies have used this approach [7]. A recent study from a demographic surveillance system (DSS) site in Malawi determined uptake of PMTCT services by linking records of DSS residents to health service data, reporting sub-optimal uptake of ART (67 % on ART during pregnancy or delivery) in the context of Option B+ [10]. High coverage (96 %) of ANC services, but only 64 % coverage with HIV testing during pregnancy, was documented within a population cohort study in Uganda [11]. No studies have provided direct estimates of PMTCT service coverage over time since the implementation of PMTCT programmes at a local level, nor the coverage at successive steps of the cascade, despite the value of such data for service monitoring. We, therefore, used data from a community cohort study in north-west Tanzania to describe and measure uptake of PMTCT services among HIV-positive pregnant women, including trends over time since the introduction of HIV and PMTCT services to the area, prior to the implementation of Option B+.

## Methods

### Setting

This study took place in Kisesa, a rural community of approximately 30,000 in north-west Tanzania, 20 km to the east of Mwanza city in Magu District. Population HIV prevalence was 6 % in women in 2011 [12] and 8 % among pregnant women during ANC surveillance across Magu district in 2006 [13].

Four government-run health facilities in Kisesa offer PMTCT services (since 2009). The health centre in the trading centre includes an ANC, voluntary counselling and testing (VCT) clinic (opened 2005), and HIV care and treatment clinic (CTC) (opened 2008). Between 2005 and 2008, pregnant women diagnosed HIV-positive at the VCT clinic were referred to Bugando hospital in Mwanza city for PMTCT services. Three dispensaries, located 5–10 km from the health centre in more rural villages, offer basic PMTCT services including HIV testing at ANC, provision of ARV prophylaxis when stocks are available, and referral to Kisesa health centre CTC. From 2008 to 2012, blood samples were transported from Kisesa CTC to Bugando hospital for CD4 count testing.

From 2005, national guidelines recommended single-dose nevirapine for mothers during labour and for infants immediately after birth, and from 2007 to 2011, ARV prophylaxis for HIV-positive pregnant women from 28 weeks gestation, during delivery and post-partum for 7 days, with infant ARV prophylaxis recommended for ≤4 weeks after birth [14] (Table 1). Women with CD4 counts <200 cells/mm^3^, clinical stage 4, or clinical stage 3 with CD4 <350 were eligible to initiate ART [14]. In 2012, the threshold for ART eligibility was raised to CD4 <350, and prophylaxis was prescribed from 14 weeks gestation until 7 days post-partum to mothers and until cessation of breastfeeding for infants (‘Option A’) [15].

**Table 1:** Summary of PMTCT interventions in Kisesa

Year of implementation	Intervention – mother (ARV prophylaxis)	CD4 count threshold for ART (cells/mm^3^)	Intervention – infant
2005	Sd-NVP during labour & delivery	CD4 <200	Sd-NVP to infants within 72 hours of birth
2007	AZT from 28 weeks gestation; sd-NVP + AZT + 3TC during labour; AZT + 3TC until 7 days postpartum	CD4 <200	NVP for 1 week after birth and AZT for up to 4 weeks
2012	‘Option A’: AZT from 14 weeks gestation; sd-NVP + AZT + 3TC during labour; AZT + 3TC until 7 days postpartum	CD4 <350	NVP until 1 week after cessation of breastfeeding (or 4–6 weeks if replacement feeding)

(sd-NVP) single-dose nevirapine; (AZT) azidothymidine; (3TC) lamivudine

### Demographic and HIV sero-surveillance

An open cohort study has been ongoing in Kisesa since 1994, including a DSS with rounds (28 to-date) of household enumeration every 4–12 months recording pregnancies, births, deaths, migrations, and 7 rounds of HIV sero-surveillance at approximately 3 year intervals (sero-survey7 in 2013). Adults aged ≥15 are eligible to participate in the sero-surveys. Participants who give their consent are tested for HIV without disclosure of results, offered VCT (since 2004), and interviewed about their use of health services. Detailed methods for the cohort were described previously [16, 17].

### Clinic data

ANC pregnancy registers for 2005–2012 and PMTCT programme registers for 2009–2012 were collected retrospectively from all four Kisesa-based facilities. Less than 10 % of records were missing. Data were double-entered into a custom-built database by trained data entry clerks at the National Institute of Medical Research in Mwanza and stored on a password-restricted computer network (PMTCT programme registers contain registration numbers: names of HIV-positive patients are not visible). CTC data are entered into a national database by government data entry clerks. Data were abstracted from 2005 to 2012 (names are stored in encrypted format), including data for Kisesa-resident patients who initially enrolled at Bugando CTC.

### Record linkage

Clinic records were linked to community cohort data with an automated matching procedure using personal attributes such as name, sex, age, and pregnancy dates. The algorithm was developed using a gold standard based on anonymous identification numbers collected from women’s ANC cards during DSS round 27. Approximately 80 % of ANC and CTC clinic records for women from Kisesa were linked to a DSS record. Positive predictive value of the algorithm was 98 %, with a sensitivity of 70 %. CTC records for women of child-bearing age were matched using a similar algorithm. Clinic records for the same woman in ANC, PMTCT or CTC registers were linked using ANC registration numbers and/or CTC numbers. Linked datasets were stored on a secure computer network, and analytical datasets were stripped of identifying information.

### Statistical analysis

Analyses were carried out using Stata12 (Stata Corp LP, Texas, USA). The denominator included pregnancies to HIV-positive women in Kisesa, resident in 2005–2012. HIV-positivity was determined from research testing during HIV sero-surveys, or linkage of a DSS record to an HIV-positive clinic record (for women whose HIV status was not known from sero-survey testing, or who had sero-converted since last testing HIV-negative). Sero-conversion dates were estimated, taking the mid-point between last HIV-negative and first HIV-positive test results for sero-incident cases. For prevalent cases, sero-conversion dates were estimated as three years prior to first testing HIV-positive at a sero-survey (based on data for sero-incident cases by sex and age). Pregnancies to HIV-positive women were identified using children’s dates of birth with links to mothers, mothers’ self-reports of recent births and pregnancies in the DSS and sero-surveys, or pregnancies recorded in the clinic. Women who were resident in Kisesa and pregnant in 2005–2012, whilst HIV-positive, were eligible for inclusion. Women who became HIV-positive after pregnancy were excluded.

The proportion of women accessing each service, per pregnancy, was calculated overall and by year of pregnancy. Service use was based on linkage of a woman’s DSS record to a clinic (ANC or CTC) record. Dates of pregnancy and clinic attendance were aligned to determine service access during a particular pregnancy. Receipt of ARV drugs during pregnancy was defined using PMTCT registers which capture ARVs dispensed during ANC, or CTC records (continuation of ART at the time of pregnancy, or initiation of ART before the recorded or estimated delivery date). PMTCT registers were also used to abstract data on gestational age at the time of enrolment into the PMTCT programme. Diagnosis of HIV before pregnancy or CTC registration was determined using attendance at VCT services in sero-surveys, or self-reported VCT use in sero-survey interviews. Diagnosis at ANC was based on linkage to HIV test results in PMTCT testing registers, or prior VCT. The proportions of women enrolling in HIV care by facility type were calculated among individuals who registered at ANC services and compared using chi square tests.

Estimates of coverage with PMTCT services (enrolment in ANC, HIV care, and receipt of ARVs during pregnancy) were adjusted for cases of clinic attendance that were missed by the linkage algorithm: raw proportions were divided by the proportion of clinic records that were linked to the DSS, or by the algorithm sensitivity (Additional file 1). Estimates were also adjusted by the proportion of women who may have accessed ANC services outside Kisesa (measured in sero-survey7 as 12 %).

Kaplan-Meier survival plots were used to analyse time to CTC registration. Time was calculated from the ANC registration date (or reported pregnancy date if ANC registration date was unknown) to the CTC registration date (or date of next visit to the CTC if already registered), or date of exit from the DSS (out-migration, death or enumerated in the latest round), or end of the study period (Dec 31 2012) if this preceded the DSS exit date. Log-rank tests were used to compare differences in time to registration by year of pregnancy.

ANC clinic records that were linked to the DSS by the matching algorithm (*n* = 9842 in total, including HIV-positive and HIV-negative women) were compared to unlinked ANC clinic records (*n* = 2579), for women documented as residing in Kisesa, in order to investigate the potential for bias. Descriptive cross-tabulations and chi-squared tests were used for this purpose. The same descriptive analyses were also conducted to compare CTC records (for Kisesa-resident women) that were linked (*n* = 661), versus not linked (*n* = 174), to DSS records.

### Ethical approval

This study was granted ethical approval by the London School of Hygiene and Tropical Medicine, and the Tanzanian Medical Research Coordinating Committee.

## Results

**Fig. 2:**
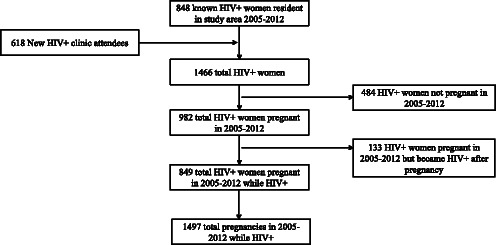
Flow chart of participants eligible for inclusion in the analysis

Eight hundred and forty eight Kisesa-resident women had ever tested HIV-positive during sero-surveillance. Another 618 DSS records were newly identified as HIV-positive based on their linkage to a record in PMTCT registers or the CTC. Of these women, 849 had been pregnant in 2005–2012 since they were infected, giving a denominator of 1497 pregnancies while HIV-positive (49 % out of 849 women had ≥2 pregnancies) (Fig. 2).

### Characteristics of the study population

Table 2 presents characteristics of included women around the time of pregnancy. Half the women were aged between 20 and 29 while pregnant, 72 % were married, 40 % were residing in remote rural villages (31 % in the trading centre, 29 % in roadside villages), and 67 % were in their third or later pregnancy (median 3rd pregnancy).

**Table 2:** Characteristics of HIV-positive women in Kisesa at the time of/closest to each pregnancy (*N* = 1497 pregnancies)

Factor	Category	Number of pregnancies	% of pregnancies
Age	<20	88	5.9
	20–29	749	50.0
	30–39	576	38.5
	40+	84	5.6
Year of pregnancy	2005	74	4.9
	2006	135	9.0
	2007	159	10.6
	2008	199	13.3
	2009	192	12.8
	2010	220	14.7
	2011	249	16.6
	2012	269	18.0
Residence area	Remote rural	600	40.1
	Roadside	434	29.0
	Trading Centre	463	30.9
Marital status	Currently married	1079	72.1
	Never married	150	10.0
	Previously married	267	17.9
Gravidity^a^	1	217	14.5
	2	275	18.4
	3	305	20.4
	4	238	15.9
	5+	462	30.9

^a^49 % of 849 women contributed ≥2 pregnancies to this analysis

### Uptake of PMTCT services

**Fig. 3:**
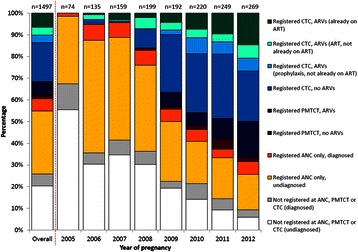
Raw proportions of pregnancies to HIV-positive women in Kisesa in which PMTCT services were accessed. *ANC* antenatal clinic, *ARV* antiretroviral, *ART* antiretroviral therapy, *CTC (HIV)* care and treatment clinic, *PMTCT* prevention of mother-to-child transmission. "Diagnosed" *[not registered at ANC, PMTCT, or CTC]* refers to women who were aware of their HIV status through VCT during earlier sero-surveys. "Diagnosed" *[registered at ANC only]* refers to women who were aware of their HIV status through earlier VCT during sero-surveys, or provider-initiated HIV testing and counselling at ANC

Figure 3 illustrates raw estimates for uptake of PMTCT service components overall, and by year of pregnancy. Of the 1497 pregnancies to (849) HIV-positive women overall, 387 (26 % out of 1497) were not linked to ANC or HIV services during their pregnancy, of whom 82 (21 % out of 387) were already diagnosed (had VCT during earlier sero-surveys). Thirty-five percent (*n* = 519/1497) registered at ANC but did not enrol in HIV services, of whom 434 (84 % out of 519; 29 % overall) had not had an HIV test at ANC or earlier VCT. A small proportion (8 %, *n* = 119/1497) enrolled in the PMTCT programme at ANC but not at the CTC: most (80 % out of 119; 6 % overall) had received ARV prophylaxis. HIV-positive women who enrolled in the PMTCT programme in 2012 had a median gestational age of 24 weeks (IQR 20–28).

Thirty-two percent (*n* = 472/1497) had registered at the CTC, of whom 204 (43 % out of 472; 14 % overall) had received ART or ARV prophylaxis during pregnancy. Overall, 20 % out of 1497 accessed ARV drugs during pregnancy at ANC or CTC. Ninety-seven (21 % out of 472, or 6 % overall) had already started ART (there were very few documented treatment interruptions, although 3 had not been seen for over 12 months, and 12 transferred to another clinic). Among 268 who registered at the CTC but did not acquire ARVs, 45 (17 % out of 268) had not attended CTC appointments for >12 months. Among those who had not started ART (*n* = 319), 48 % had no CD4 result recorded, while 11 % were eligible for ART.

Prior to 2008, few individuals were enrolled in HIV services during pregnancy. Uptake of PMTCT services increased over time, with the largest increases occurring between 2007 and 2008 (opening of Kisesa CTC) and between 2008 and 2009 (implementation of the PMTCT programme in Kisesa). In 2009, 44 % (*n* = 85/192) had enrolled in the CTC or ANC-based PMTCT services, and 17 % (*n* = 32/192) had been prescribed ARV prophylaxis or ART. These proportions increased to 68 % (*n* = 184/269) in care and 44 % (*n* = 117/269) accessing ARVs in 2012 (raw estimates). Among those registered at the CTC in 2012 (*n* = 134), 39 (29 %, or 14 % out of 269) had already started ART. Eighteen women eligible for ART in 2012 had not started (23 % out of 78 CTC patients not on ART, versus 9 % in 2009).

**Fig. 4:**
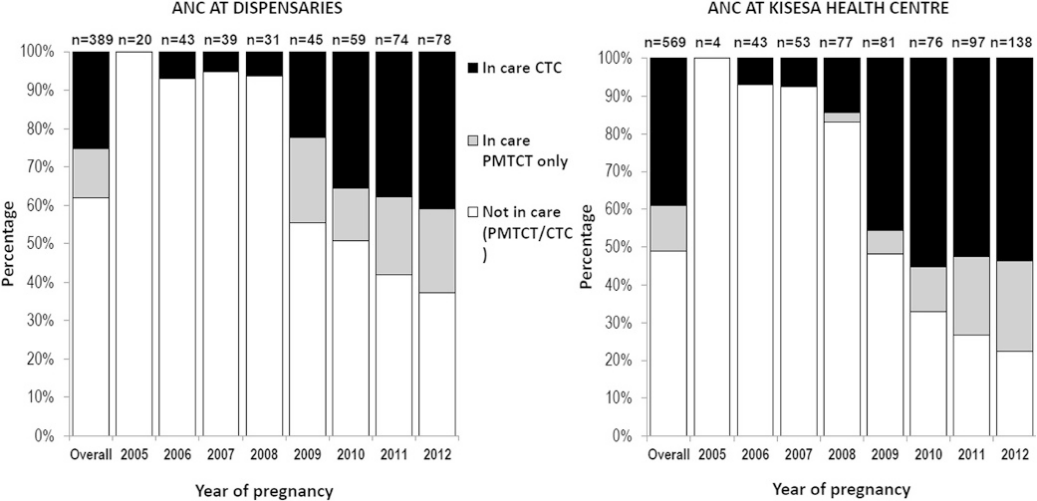
Access to HIV care, per pregnancy, among HIV-positive women enrolled in Kisesa ANC (health centre/dispensaries). *ANC* antenatal clinic, *CTC* care and treatment clinic, *PMTCT* prevention of mother-to-child transmission

Women who attended ANC at dispensaries (*n* = 389 pregnancies) were less likely to be in care (*n* = 148, 38 %) than women who attended ANC at the health centre (51 % out of 569 pregnancies) (*p* <0.001), with the differential remaining over time (Fig. 4).

### Coverage estimates

Adjusted coverage estimates for the proportion of HIV-positive women who accessed ANC, PMTCT/CTC (HIV care), and ARVs per pregnancy in 2005–2012 are summarised in Table 3. The (adjusted) proportion of HIV-positive women accessing ANC was estimated to be as high as 92 %, 57 % for access to HIV care, and 29 % for coverage with ARV drugs. By 2012, coverage with HIV care was estimated to reach >90 % while coverage with ARVs reached 62 % (calculations in Additional file 1).

**Table 3:** Coverage estimates (raw/adjusted) for the proportion of HIV-positive women accessing service components per pregnancy (2005–2012)

Service	Raw estimate	Estimate adjusted by % ANC records linked to DSS*	Estimate adjusted by % ANC records linked to DSS & local ANC attendance**	Estimate adjusted by algorithm sensitivity***
ANC	64.0 %^a^ [61.6–66.4 %]	80.6 %	91.6 %	91.4 %
In HIV care (PMTCT/CTC)	39.5 % [37.0–42.0 %]	49.7 %	56.5 %	56.4 %
ARV drugs	20.0 % [17.9–22.0 %]	25.2 %	28.6 %	28.6 %

*ANC* antenatal clinic, *ARV* antiretroviral, *CTC* care and treatment clinic, *DSS* demographic surveillance system, *PMTCT* prevention of mother-to-child transmission

*raw estimate divided by 79 % (% of ANC records linked to the DSS)

**previous estimate (column three) divided by 88 % (accounting for 12 % of women that reported accessing ANC services outside Kisesa ward in sero7)

***divided by 70 % (sensitivity of the algorithm)

^a^ raw estimate for the proportion who attended ANC, excluding women who attended CTC in Kisesa but were not linked to Kisesa ANC services (e.g., may have attended ANC outside the area)

### Time to CTC visit

**Fig. 5:**
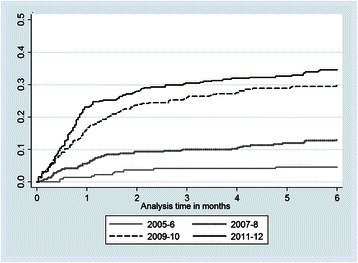
Kaplan Meier plot of time to CTC visit by year of pregnancy

Figure 5 illustrates the time to CTC visit by calendar year of pregnancy over the first six months of follow-up. Time to CTC visit decreased with increasing year of pregnancy (*p* <0.001). For pregnancies since 2009 a steeper increase was noticeable within the first month compared to earlier years, corresponding to a greater proportion of women visiting the CTC within this time window.

### Characteristics of clinic attendees by linkage to the DSS

Compared to ANC records that were linked to the DSS (*n* = 9842), unlinked (Kisesa-resident) ANC records (*n* = 2579) were more often from a dispensary (44 % versus 39 %, *p* <0.001) (data not shown), but there was no difference in age or year of registration. A greater proportion of unlinked CTC records (*n* = 174) were registrations prior to 2008 (25 %, compared to 3 % of linked CTC records (*n* = 661), *p* <0.001), but there was no difference in age, village of residence, clinical stage or CD4 count at registration.

## Discussion

This study documented fairly low population-level uptake of PMTCT services in rural Tanzania in 2005–2012 before the implementation of Option B+. However, primary care ART and PMTCT services were only introduced in Kisesa in 2008–9, after which there was an encouraging upward trend in service use over time; coverage with HIV care during pregnancy rose to 68 % (raw; >90 % adjusted), and with ARV drugs to 44 % (raw; 62 % adjusted) in 2012. These estimates are lower than national-level estimates of coverage with ARVs for HIV-positive pregnant women in Tanzania (73 % in 2013 [2]), potentially reflecting over-estimates at the national and regional levels [7], and/or local level differences. ANC surveillance across Magu District in 2008 identified very low usage of ARVs (33 %) for PMTCT based on self-reports by pregnant women [18]. Qualitative research in Kisesa and other African studies have identified many factors at the level of individuals (e.g., psychological issues following diagnosis, and poor knowledge of HIV), the community (e.g., stigma, fear of disclosing HIV status, and the amount of support from partners or relatives) and health-systems (e.g., accessibility of services, staff shortages, and interactions between patients and health care providers) that may contribute to poor uptake of PMTCT services [19, 20]. A quantitative analysis also revealed that marital status (currently married), increasing age, residence in roadside areas, later calendar time period (year of pregnancy) and increasing infection duration were associated with greater access to HIV care and/or ARV drugs for PMTCT in Kisesa [21].

Weaknesses in the PMTCT programme were evident throughout the cascade, but were notable at the point of testing, assessment for ART eligibility and receipt of ART or prophylaxis. The adjusted estimate for coverage with ANC was within the range of survey data for Mwanza region in 2010 (86 %) [22] and Kisesa (98 % in 2010 (sero-survey6); 90 % in 2013 (sero-survey7)). Our analysis also identified some HIV-positive women who were aware of their status through earlier VCT but did not attend health services during pregnancy, calling for strengthened post-test counselling about the importance of PMTCT.

The sizeable proportion of HIV-positive women who were not enrolled in PMTCT or CTC services during pregnancy was primarily accounted for by women who attended ANC but were not diagnosed (were not tested at ANC and did not have earlier VCT). This is most likely explained by frequent and persistent stock-outs of HIV test kits during the study time-frame [19, 23]. Low coverage (64 %) with HIV testing at ANC was also documented in Uganda in 2008–2010 [11], while Tanzanian programme statistics indicate that 20 % of women attending ANC were not tested in 2011 [24]. Some women may decline testing, fearing disclosure of HIV-positive results and consequent conflict in relationships [19, 25]. Improvements in the distribution of HIV test kits, community-level interventions to reduce HIV stigma, as well as adequate pre-test counselling (sometimes omitted [23]) may help to reduce drop-outs at this stage.

Most women who enrolled in the PMTCT programme received ARV prophylaxis and were therefore covered with the minimum PMTCT intervention. However, this pre-supposes (for maximum efficacy) optimal adherence and initiation of ARVs from the recommended gestational age, while further analyses of PMTCT clinic records revealed that women were registering late (median 24 weeks (IQR 20–28) in 2012). As Tanzania rolls out Option B+, it will be important to emphasise the importance of attending ANC early, and to monitor the subgroup that registers for PMTCT but not CTC or ART services. Research from Malawi indicates that some pregnant women may avoid ART, for example because they feel in good health [10]. Other reasons for not taking up ART in the context of Option B+ may include anxiety about the prospect of taking treatment for life, feeling overwhelmed, as well as more general barriers to ART including facility accessibility, stigma, fear of disclosure, and limited knowledge of ART [26].

Access to CTC services, including the time to CTC attendance, improved each year following the availability of ART and PMTCT services in Kisesa, but remained a point of further attrition. Linkage to HIV care and treatment has been highlighted as a problematic step in the PMTCT programme [27], including in Mwanza city [28]. Starting ANC at dispensaries was apparently a disadvantage, reflecting the need for referrals to the CTC and associated accessibility issues [19]. Further decentralisation of ART services would help in this regard and will be crucial to the success of Option B+. Improvements in the design, usage and storage of PMTCT transfer forms may also strengthen linkages between ANC and CTC [29].

A considerable weakness was the uptake of ARV drugs among those registered for CTC. Almost a quarter of women eligible for ART in 2012 had not received it, potentially reflecting adjustment to new (widened) eligibility criteria. Losses to follow-up were also partly responsible for not initiating treatment, while half the women who had not started ART had no CD4 count documented. Limited CD4 count testing was also reported in PMTCT programmes in Mwanza city hospitals [28]. Implementation of Option B+ will simplify decisions regarding ART initiation for HIV-positive pregnant women by eliminating the need to assess treatment eligibility through CD4 counts. Point of care CD4 count tests have recently been introduced and should improve long-term monitoring of immunological status. Nonetheless, women without CD4 counts or ineligible for ART should have received ARV prophylaxis. Documentation of prophylaxis in the CTC database was weak, with additional hand-written notes non-systematically present in patient files. The proportion receiving prophylaxis may therefore be under-estimated, highlighting the importance of robust and systematic approaches to routine data capture [7].

The increasing proportion of women who were already on ART before pregnancy (14 % in 2012) is noteworthy, as this population presents new challenges for PMTCT programmes, including monitoring of treatment adherence and virologic suppression [30], and will grow with Option B+.

Linkage of community cohort and clinic data was the key strength and novelty of this analysis, providing estimates of population-level coverage with PMTCT services, although there were inherent limitations. The proportion of clinic records that was not linked to the DSS was accounted for, but was a potential source of bias given differences observed in the characteristics of linked and unlinked clinic records: uptake of services before 2008 or in the dispensaries may be under-estimated, although this is unlikely to have altered our conclusions. Routine clinic data were abstracted retrospectively from health facility registers, contributing to general limitations in the data quality. Poor quality of some clinic data (e.g., missing or duplicate identifiers) complicated the linkage of records from different registers and tracking of women who had switched facilities [7], potentially under-estimating the proportions tested and enrolled in care, although mis-matches are likely to have resulted in random misclassification of outcomes. The large proportion (approximately 20 %) of delivery registers missing ANC numbers and difficulties linking infant and maternal records also restricted our analysis to PMTCT service components during pregnancy. Weaknesses in facility record-keeping may have partly explained low coverage of ARV prophylaxis. Estimated HIV sero-conversion dates or self-reported pregnancy dates may be inaccurate, and HIV-positive pregnancies may have been erroneously included in or omitted from the denominator, leading to under- or over-estimates of coverage respectively.

## Conclusion

In conclusion, population-level uptake of PMTCT services in this rural Tanzanian setting was disappointingly low and below national-level estimates, but improved markedly over time. Given the proportion of women who fully adhere to regimens and the proportion of mothers *and* infants receiving ARVs is likely to be even lower, the number of infants potentially at risk of acquiring HIV is a concern. Implementation of Option B+ is likely to simplify decisions for initiating HIV-positive pregnant women onto ART, while further decentralisation of CTC services and careful management of stocks of HIV test kits will be key to overcoming other weaknesses in the PMTCT programme.

## Additional file

Additional file 1: **Adjusted coverage estimates for the proportion of HIV-positive pregnant women who a) accessed ANC, b) enrolled in care at PMTCT/CTC, and c) accessed ARVs, in each pregnancy by year.** (XLSX 13 kb)

## Abbreviations

ANC: antenatal clinic
ART: antiretroviral treatment
ARV: antiretroviral
CD4: cluster of differentiation 4 positive T cells
CTC: care and treatment clinic
DSS: demographic surveillance system
HIV: human immunodeficiency virus
PITC: provider-initiated testing and counselling
PMTCT: prevention of mother-to-child transmission
VCT: voluntary counselling and testing
